# Neurobiological modulation with REAC technology: enhancing pain, depression, anxiety, stress, and quality of life in post-polio syndrome subjects

**DOI:** 10.1038/s41598-024-68200-5

**Published:** 2024-07-26

**Authors:** Jeyce Adrielly André Nogueira, Acary Souza Bulle Oliveira, Monalisa Pereira Motta, Alcione Aparecida Vieira de Souza Moscardi, Vanessa Manchim Favaro, Claudete Munhoz Teixeira, Amanda Orasmo Simcsik, Maria Clara Patrizi, Maria Salete Conde, Arianna Rinaldi, Vania Fontani, Salvatore Rinaldi

**Affiliations:** 1 Division of Neuromuscular Diseases, Department of Neurology and Neurosurgery, Federal University of São Paulo, São Paulo, 01000-000 Brazil; 2 Department of Preventive Medicine, Federal University of São Paulo, São Paulo, 01000-000 Brazil; 3 Department of Adaptive Neuro Psycho Physio Pathology and Neuro Psycho Physical Optimization, Rinaldi Fontani Institute, 50144 Florence, Italy; 4 Department of Regenerative Medicine, Rinaldi Fontani Institute, 50144 Florence, Italy; 5 Research Department, Rinaldi Fontani Foundation, 50144 Florence, Italy

**Keywords:** Post-polio syndrome, REAC technology, Pain, Depression, Anxiety, Stress, Quality of life, Quality of life, Neurological disorders

## Abstract

Post-polio syndrome (PPS) brings new challenges for polio survivors, including muscle decline, pain, depression, and diminished quality of life. This study explored the potential of REAC neuromodulatory treatments to ease pain, improve mood, and enhance quality of life in PPS patients. 17 individuals with PPS (average age 54.8) received three REAC treatments: Neuro Postural Optimization, Neuro Psycho Physical Optimization, and Neuro Psycho Physical Optimization-Cervico Brachial. Pain, depression, anxiety, stress, and quality of life were assessed before and after using established scales. REAC treatments significantly reduced pain across various dimensions, along with depression, anxiety, and stress levels. Additionally, patients reported improved physical and psychological quality of life. This study suggests REAC neuromodulatory treatments as a promising non-invasive option to improve pain, emotional well-being, and quality of life in individuals with PPS.

## Introduction

Post Polio Syndrome^1^ (PPS) is a complex and debilitating condition that affects a significant number of individuals who have previously recovered from acute poliomyelitis. Characterized by a progressive decline in muscle function and new-onset symptoms after years of stability, PPS poses considerable challenges to the affected individuals, impacting their overall quality of life^2^. Among the numerous factors contributing to the burden of PPS, psychological [SHIRI 2015] and emotional well-being^3^ have emerged as crucial aspects that warrant comprehensive examination.

Individuals with physical conditions often experience heightened levels of emotional distress and psychiatric symptoms, which can exacerbate physical symptoms and complicate disease management^4^. Individuals with Post-polio Syndrome have reported higher levels of emotional distress compared to the general population^5^.

The intricate interplay between these psychological factors and their consequences on the overall health and well-being of PPS patients calls for a thorough exploration to improve clinical interventions and enhance patients' quality of life^4,5^.

Pain, a predominant feature of PPS^6^, not only impacts the physical discomfort experienced by individuals but also intertwines with psychological states. The relationship between pain and emotional well-being is complex, with chronic pain often leading to depression, anxiety, and stress, while these psychological states, in turn, can amplify the perception of pain. Understanding the correlations between pain and mental health in the context of PPS is crucial in developing multidimensional approaches that address both physical and psychological aspects of this condition^7^.

The quality of life is determined by multiple factors, including physical and mental aspects, as well as other factors such as hope and employment^8^. Patients with PPS face various challenges that affect their well-being, in addition to physical limitations. Elucidating the multifaceted impact of depression, anxiety, stress, and pain on the quality of life in PPS is essential for the development of comprehensive care strategies that holistically address the diverse needs of these patients^9^.

This manuscript aims to present a potential therapeutic strategy based on some neurobiological modulation treatments using Radio Electric Asymmetric Conveyer (REAC) technology. The utilized treatments are referred to as Neuro Postural Optimization (NPO)^10,11^, Neuro Psycho Physical Optimization (NPPO)^12^, and Neuro Psycho Physical Optimization—Cervico Brachial (NPPO-CB)^13^, Neuro Muscular Optimization (NMO) with the goal of ameliorating mood and behavioral disorders, as well as psychogenic pain^14^. Their utility in this patient population was evaluated through three specific tests assessing Pain, Depression, Anxiety, Stress, and Quality of Life.

## Results

The sample consisted of 17 participants aged between 48 and 63 years. The observed mean age was 54.88 years (± 4.47 years), with a median of 55 years. Of the total sample, 12 participants (70.6%) reported identifying as female, while 5 participants (29.4%) indicated identifying as male. The PPS diagnosis was based on Halstead criteria, validated by a college of international experts^15^. Other potential medical or surgical causes that could be responsible for the non-specific new symptoms, before validating the PPS diagnosis, were excluded after careful clinical and laboratory evaluation. The patients were undergoing regular outpatient follow-up, with symptomatic therapeutic guidance. Clinical manifestations had been stable over the last 4 months. During the intervention phase, the only therapeutic modification was the introduction of REAC.

### McGill pain assessment (McGill)

**Figure 1:**
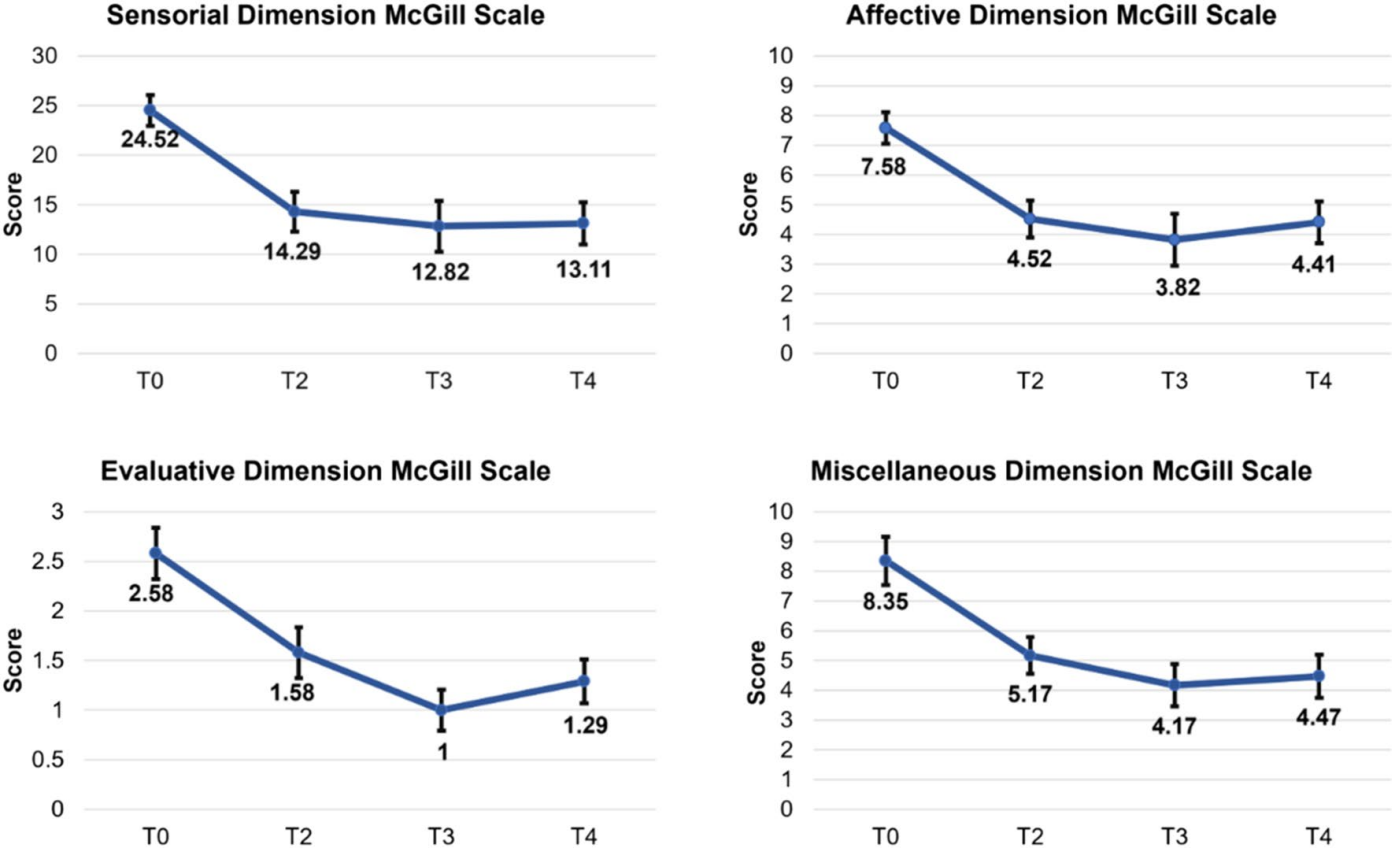
The figure displays the means of the scores for the sensory, affective, evaluative and miscellaneous dimensions of the McGill Pain Test in the T0 (N = 17), T2 (N = 17), T3 (N = 17), and T4 (N = 17) conditions.

At T0, a pain index of 43.05 was observed, which reduced to 23.29 at the last evaluation. The initial pain level, assessed using the Visual Analogue Scale, was 3.0, which decreased to 1.17 at T4. A repeated-measures analysis of variance (ANOVA-RM) was conducted to compare the McGill scores across four distinct conditions: T0, T2, T3, and T4. The ANOVA-RM revealed statistically significant differences in multiple McGill dimensions among these conditions. Specifically, the sensory (F(3, 48) = 16.03, *p* < 0.0001; ƞ^2^ = 0.50), affective (F(3, 48) = 10.39, *p* < 0.0001; ƞ^2^ = 0.39), evaluative (F(3, 48) = 9.21, *p* < 0.0001; ƞ^2^ = 0.36), and miscellaneous (F(3, 48) = 10.63, *p* < 0.0001; ƞ^2^ = 0.39) dimensions showed significant variations. To further elucidate these differences, post hoc analyses using the Bonferroni correction further confirmed these differences, as summarized in Table 1, and depicted in Fig. 1.

**Table 1:** Results of post-hoc comparisons among conditions in the McGill Pain Questionnaire and Visual Analogue Scale.

Condition	Sensory	*p* value
T0 (24.52 ± 6.37)	T2 (14.29 ± 8.27)	< 0.0001*
	T3(12.82 ± 10.51)	< 0.0001*
	T4 (13.11 ± 8.80)	< 0.0001*
T2	T3	> 0.05
	T4	> 0.05
T3	T4	> 0.05

Condition	Affective	*p* value
T0 (7.58 ± 2.20)	T2 (4.52 ± 2.57)	0.002*
	T3 (3.82 ± 3.59)	0.001*
	T4 (4.41 ± 2.89)	0.010*
T2	T3	> 0.05
	T4	> 0.05
T3	T4	> 0.05

Condition	Evaluative	*p* value
T0 (2.58 ± 1.06)	T2 (1.58 ± 1.06)	0.061
	T3 (1.00 ± 0.86)	0.001*
	T4 (1.29 ± 0.91)	0.015*
T2	T3	> 0.05
	T4	> 0.05
T3	T4	> 0.05

Condition	Miscellaneous	*p* value
T0 (8.35 ± 3.35)	T2 (5.17 ± 2.55)	0.022*
	T3 (4.17 ± 2.92)	< 0.0001*
	T4 (4.47 ± 2.98)	0.010*
T2	T3	> 0.05
	T4	> 0.05
T3	T4	> 0.05

Condition	McGill total pain index	*p* value
T0 (43.05 ± 9.76)	T2 (25.58 ± 12.85)	< 0.0001*
	T3 (21.82 ± 16.84)	< 0.0001*
	T4 (23.29 ± 15.05)	0.001*
T2	T3	> 0.05
	T4	> 0.05
T3	T4	> 0.05

Condition	Visual analogue scale	*p* value
T0 (3.0 ± 0.79)	T2 (1.47 ± 1.12)	< 0.0001*
	T3 (1.64 ± 1.27)	0.001*
	T4 (1.17 ± 1.18)	< 0.0001*
T2	T3	> 0.05
	T4	> 0.05
T3	T4	> 0.05

Mean and standard deviation (Mean ± SD) for each condition is presented in parentheses. * Indicates statistical significance (*p* <  0.05).

Significant values are in bold.

These findings suggest that the NPO, NPPO, and NMO protocols may have differentially influenced pain perception (Table 1 and in Fig. 1). A difference in the frequency and type of descriptors mentioned by patients was observed in each phase of the research. Table 2 displays the most selected descriptors.

**Table 2:** Most frequent descriptors chosen in each phase on the McGill Pain Scale.

	T0	T2	T3	T4
Sensory	Throbbing (77%)	Sharp (65%)	Jumping (53%)	Pricking (65%)
	Sharp (65%)	Tugging (65%)	Sharp (47%)	Tugging (65%)
	Tugging (65%)	Jumping (53%)		Sharp (53%)
	Burning (59%)			Jumping (53%)
	Jumping (53%)			
	Pricking (53%)			
Affective	Wretched (71%)	Sickening (71%)	Tiring (65%)	Sickening (82%)
	Exhausting (65%)	Wretched (65%)	Sickening (59%)	Wretched (71%)
	Sickening (65%)	Tiring (65%)	Annoying (47%)	Tiring (65%)
	Punishing (59%)	Punishing (65%)	Punishing (59%)	
	Fearful (53%)	Annoying (41%)		Fearful (53%)
Evaluative	Troublesome (29%)			Annoying (47%)
	Miserable (29%)			
Miscellaneous	Nagging (59%)	Nagging (59%)	Nagging (59%)	Nagging (59%)
	Drawing (47%)	Cool (47%)	Cool (53%)	

### Depression, anxiety and stress scale 21 (DASS-21)

The Depression, Stress and Anxiety Scale (DASS-21) was used to assess the participants' mental health. At the beginning of the study, 41.2% reported experiencing depression, 64.7% reported anxiety, and 76.5% reported stress. Moderate depression was observed in 17.6% of participants at T0, but this decreased to 0% at T4. At the start of the research (T0), 23.5% of participants exhibited extremely intense anxiety, which decreased to 6.3% by the end of the study (T4). Similarly, 29.4% of participants reported severe stress at T0, but this level of stress was absent at T4. Tables 2, 3, and 4 provide the distribution of participants among the five severity levels.

**Table 3:** Distribution between the five severity levels from the DASS-21 test in the T0 (N = 16), T2 (N = 16), T3 (N = 16), and T4 (N = 16) conditions.

	T0	T2	T3	T4
Depression DASS-21				
Normal	10 (58.8%)	14 (82.4%)	16 (94.1%)	14 (82.4%)
Mild	1 (5.9%)	0 (0%)	0 (0%)	1 (5.9%)
Moderate	3 (17.6%)	2 (11.8%)	1 (5.9%)	1 (5.9%)
Severe	2 (11.8%)	1 (5.9%)	0 (0%)	0 (0%)
Extremely severe	1 (5.9%)	0 (0%)	0 (0%)	0 (0%)
Anxiety DASS-21				
Normal	6 (35.3%)	9 (52.9%)	12 (70.6%)	13 (81.3%)
Mild	1 (5.9%)	3 (17.6%)	1 (5.9%)	1 (6.3%)
Moderate	6 (35.3%)	5 (29.4%)	3 (17.6%)	0 (0%)
Severe	0 (0%)	0 (0%)	1 (5.9%)	1 (6.3%)
Extremely severe	4 (23.5%)	0 (0%)	0 (0%)	1 (6.3%)
Stress DASS-21				
Normal	4 (23.5%)	7 (41.2%)	10 (58.8%)	13 (81.3%)
Mild	6 (35.3%)	5 (29.4%)	2 (11.8%)	0 (0%)
Moderate	1 (5.9%)	3(17.6%)	2 (11.8%)	4 (25%)
Severe	5 (29.4%)	2 (11.8%)	1 (5.9%)	0 (0%)
Extremely severe	1 (5.9%)	0 (0%)	2 (11.8%)	0 (0%)

**Table 4:** Results of post-hoc comparisons among conditions in the dimensions of the DASS-21 questionnaire.

Condition	Depression	*p* value
T0 (9.50 ± 8.04)	T2 (5.37 ± 4.71)	> 0.05
	T3(4.25 ± 4.61)	0.073*
	T4 (3.25 ± 4.78)	0.094*
T2	T3	> 0.05
	T4	> 0.05
T3	T4	> 0.05

Condition	Anxiety	*p* value
T0 (11.50 ± 10.54)	T2 (6.37 ± 4.63)	> 0.05
	T3 (4.75 ± 5.00)	0.029*
	T4 (4.37 ± 6.11)	> 0.05
T2	T3	> 0.05
	T4	> 0.05
T3	T4	> 0.05

Condition	Stress	*p* value
T0 (18.50 ± 7.71)	T2 (13.37 ± 7.99)	> 0.05
	T3 (13.75 ± 12.95)	> 0.05
	T4 (9.62 ± 7.80)	0.007*
T2	T3	> 0.05
	T4	> 0.05
T3	T4	> 0.05

Mean and standard deviation (Mean ± SD) for each condition is presented in parentheses. * Indicates statistical significance (*p* < 0.05).

**Figure 2:**
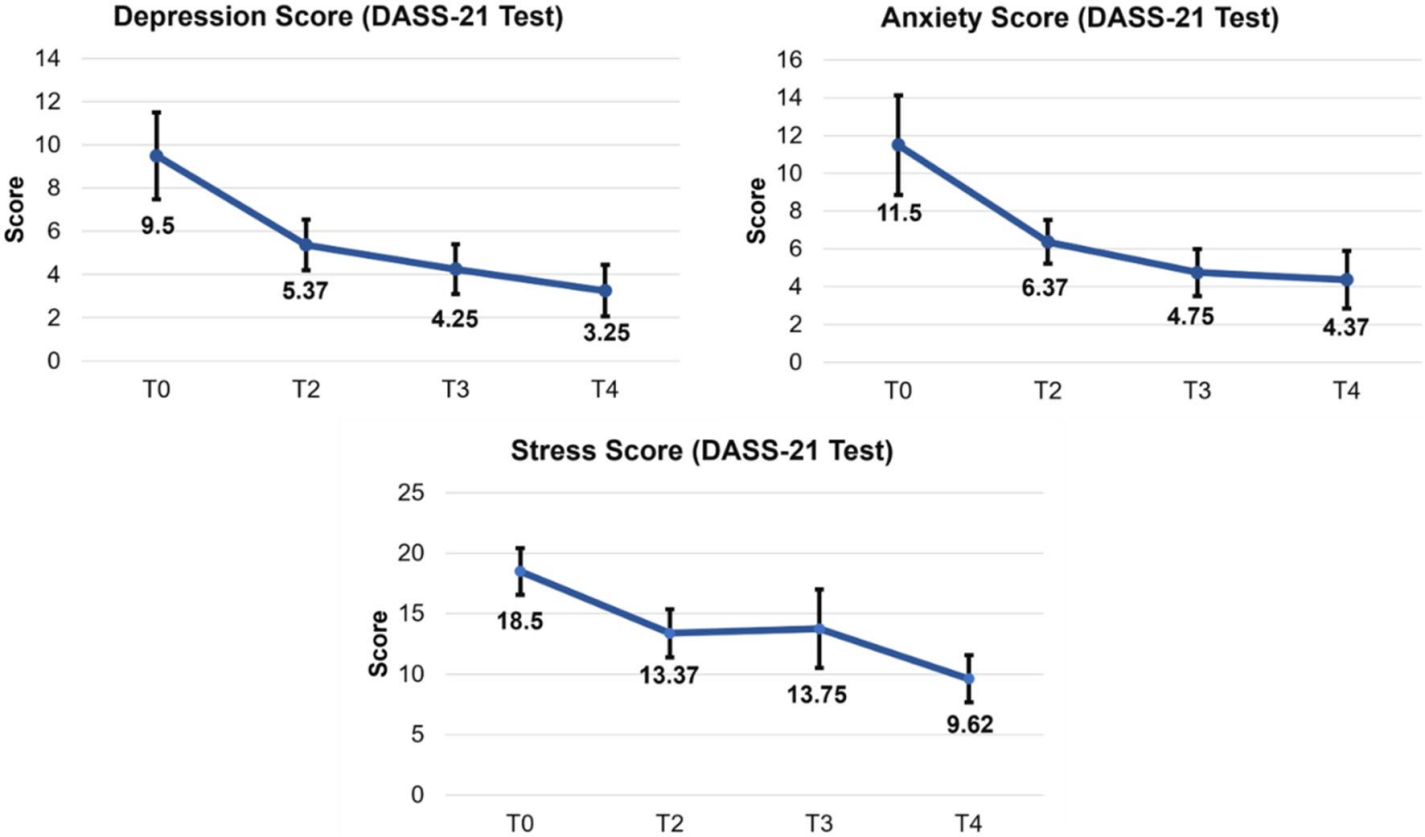
The figure displays the means of depression, anxiety and stress scores from the DASS-21 test in the T0 (N = 16), (2) T2 (N = 16), T3 (N = 16), and T4 (N = 16) conditions.

A Repeated Measures Analysis of Variance (ANOVA-RM) was performed to compare the scores on the DASS-21 assessment across four conditions: T0, T2, T3, and T4 (after NMO treatment). The analysis revealed statistically significant differences among the four conditions for depression (F(1.68, 25.27) = 5.88, *p* = 0.01; ƞ^2^ = 0.28), anxiety (F(1.45, 21.83) = 5.79, *p* = 0.01; ƞ^2^ = 0.27), and stress (F(3, 45) = 4.25, *p* = 0.01; ƞ^2^ = 0.22). Subsequent to the primary analysis, Bonferroni post-hoc assessments were conducted to identify the specific differences among the conditions. These analyses revealed that participants had significantly higher DASS-21 scores for depression, anxiety, and stress in the T0, T2, and T3 conditions compared to the T4 (see Table 3 and 4, Fig. 2).

### World Health Organization quality of life—abbreviated (WHOQoL-Bref)

**Figure 3:**
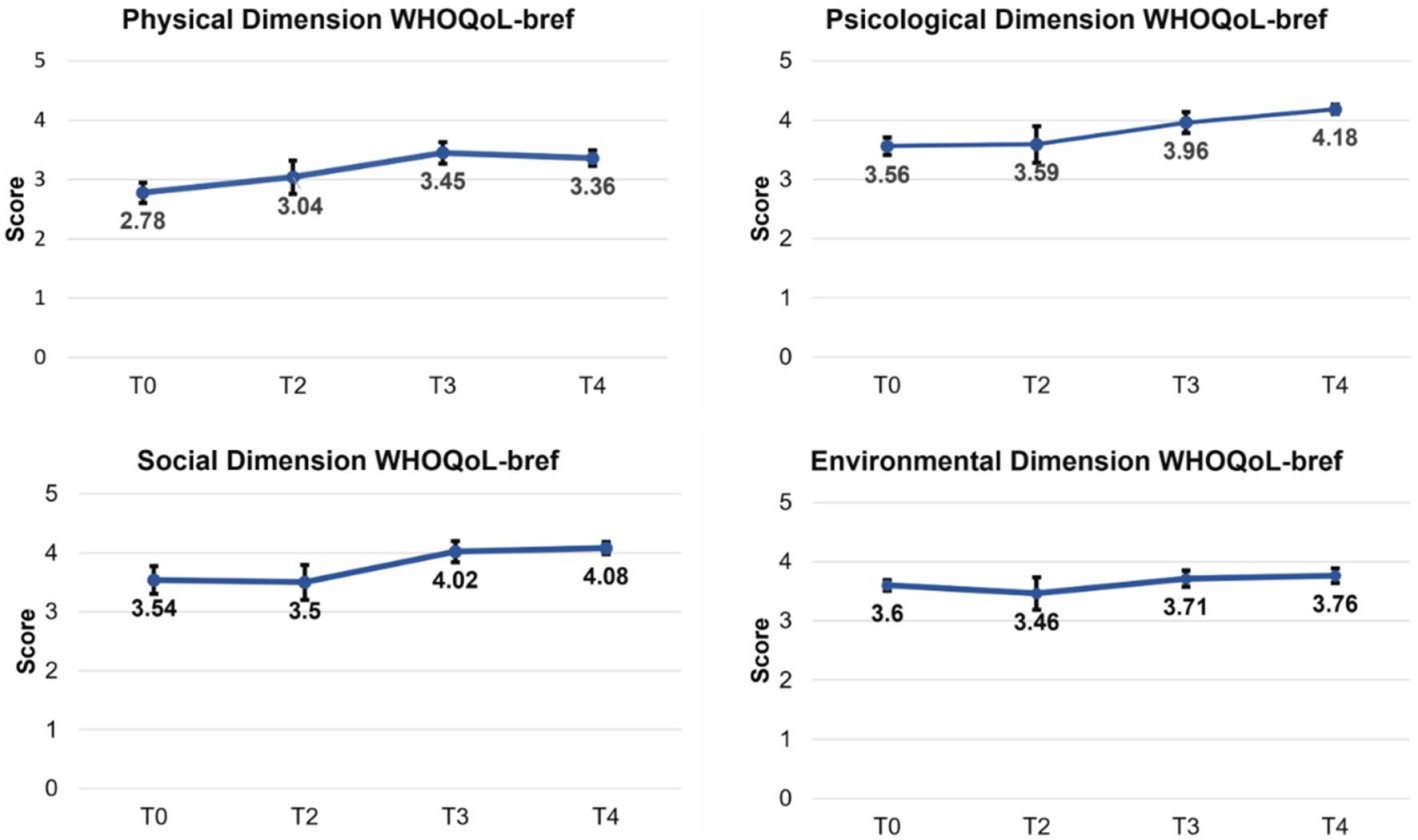
The figure shows the mean scores of the physical, phycological, social and environmental dimensions of the WHOQoL-bref test in the following conditions: T0 (N = 15), T2 (N = 15), T3 (N = 15), and T4 (N = 15).

The ANOVA-RM was conducted to compare the scores on the WHOQoL-bref) assessment across four conditions: T0, T2, T3 and T4. The ANOVA-RM revealed statistically significant differences among the physical functioning (F (1.78, 24.98) = 4.35, *p* = 0.02; ƞ^2^ = 0.23) psychological well-being (F (1.90, 26.72) = 3.30, *p* = 0.05; ƞ^2^ = 0.19), social relationships (F (3,42) = 3.22, *p* = 0.03; ƞ^2^ = 0.18), and health satisfaction (F (3,42) = 3.25; *p* = 0.031; ƞ^2^ = 0.18). After the primary analysis, Bonferroni post-hoc assessments were conducted to identify the specific differences among the conditions. These analyses revealed that participants had significantly higher WHOQoL-Bref scores in the T0, T2, and T3 conditions compared to the T4 (see Table 5 and Fig. 3).

**Table 5:** Results of post hoc comparisons between conditions in the dimensions of the WHOQoL-Bref test.

Condition	Physical	*p* value
T0 (2.78 ± 0.66)	T2 (3.04 ± 1.08)	> 0.05
	T3 (3.45 ± 0.70)	< 0.0001*
	T4(3.36 ± 0.51)	0.015*
T2	T3	> 0.05
	T4	> 0.05
T3	T4	> 0.05

Condition	Psychological	*p* value
T0 (3.56 ± 0.58)	T2 (3.59 ± 1.19)	> 0,05
	T3 (3.96 ± 0.70)	0.039*
	T4 (4.18 ± 0.31)	0.016*
T2	T3	> 0.05
	T4	> 0.05
T3	T4	> 0.05

Condition	Social	*p* value
T0 (3.54 ± 0.90)	T2 (3.50 ± 1.14)	> 0.05
	T3 (4.02 ± 0.69)	> 0.05
	T4 (4.08 ± 0.39)	> 0.05
T2	T3	> 0.05
	T4	> 0.05
T3	T4	> 0.05

Condition	Environmental	*p* value
T0 (3.60 ± 0.35)	T2 (3.46 ± 1.06)	> 0.05
	T3 (3.71 ± 0.54)	> 0.05
	T4 (3.76 ± 0.48)	> 0.05
T2	T3	> 0.05
	T4	> 0.05
T3	T4	> 0.05

Condition	Health perception	*p* value
T0 (3.33 ± 1.11)	T2 (3.66 ± 1.11)	> 0.05
	T3 (4.00 ± 0.65)	> 0.05
	T4 (4.00 ± 0.53)	> 0.05
T2	T3	> 0.05
	T4	> 0.05
T3	T4	> 0.05

Condition	Health satisfaction	*p* value
T0 (2.53 ± 1.40)	T2 (3.33 ± 1.17)	> 0.05
	T3 (3.33 ± 1.23)	> 0.05
	T4 (3.73 ± 0.79)	0.042*
T2	T3	> 0.05
	T4	> 0.05
T3	T4	> 0.05

Mean and standard deviation (Mean ± SD) of each condition is presented in parentheses. * Statistically significant (*p* < 0.05). * Indicates statistical significance (*p* < 0.05).

Significant values are in bold.

## Discussion

The emergence of new PPS symptoms can result in functional decline in this population, which can be problematic when combined with previous sequelae. Muscle and joint pain are among the three most common symptoms in PPS that interfere with the performance of daily activities, as well as practical and occupational life, directly affecting quality of life^16–18^.

In the present study, all participants reported some level of pain, quantified as an average of 3 ± 0.79 on a 0–5 pain scale at baseline. However, in this study, pain was objectively measured using a standardized pain scale. Pain can become disabling if left untreated, negatively impacting the patient’s physical and mental well-being, and interfering with their daily activities and quality of life. As pain is influenced by cultural, emotional, and environmental factors, it has traditionally been seen as a subjective experience. Werhagen et al.^19^ evaluated the impact of pain on the quality of life of patients with PPS. The study found that patients who reported pain had worse quality of life in the SF-36 subdomains Body Pain (*p* < 0.001), General Health Status (*p* < 0.005), Vitality (*p* < 0.032), and for the Physical Compound Score (PCS) (*p* < 0.002) compared to those who did not report pain on clinical examination^19^.

The present study utilized the McGill scale to assess symptoms in multiple domains, both quantitatively and qualitatively. The results indicate a statistically significant reduction in pain scores across all dimensions (sensory, affective, evaluative, and miscellaneous) following the application of the REAC protocols, compared to scores obtained prior to treatment (T0). Additionally, there were qualitative changes in pain perception, as evidenced by the reduction and variation of the McGill scale descriptors. In the sensory-discriminative dimension, which pertains to the mechanical, thermal, and spatial properties of pain^20^, the most common descriptors at T0 were ‘sharp’ and ‘pull’, which were replaced by ‘stinging’ at T4. The group associated with affective-motivational responses in individuals is linked to tension, anxiety, and neurovegetative responses^20^. The most frequent descriptor at T0 was ‘miserable’, while at T4 it was ‘disgusting’. ‘Problematic’ and ‘miserable’ were the most used words for the cognitive-evaluative component at T0, while at T4 it was ‘boring’. The miscellaneous dimension showed no change in the most frequent descriptor, which was ‘annoying’ at both T0 and T4. This presentation of descriptors is crucial for a qualitative assessment of pain and can assist in therapeutic intervention by providing a better definition^20^. These results indicate a broad impact of REAC protocols on participants’ pain perception.

Supporting these findings, a randomized, blinded, placebo-controlled study of 888 patients with clinically unexplained symptoms and presenting pain and physical problems showed a statistically significant reduction in both aspects after completing a cycle of NPPO^21^. Additionally, the intervention group exhibited a statistically significant reduction in the Psychological Stress Measure test score. According to the study, REAC technology is a non-drug and non-invasive alternative for treating pain, particularly in cases where emotional state and stress levels significantly impact overall symptoms^21^.

Regarding PPS, there has been much discussion about the physical aspects of the disease and the limitations it imposes on patients. It is worth noting that the emotional aspects of individuals diagnosed with post-polio syndrome can be influenced by their life history, as well as the clinical and social challenges faced during childhood. It is important to highlight that the care required for this condition can trigger children's fears and suffering^22^. Shiri et al.^5^ found that emotional distress was higher in the polio sample than in the general population^5^.

The study assessed emotional state and stress levels using the DASS-21. Prior to treatment, 41.2% of participants reported experiencing some level of depression. The study was conducted during the COVID-19 pandemic, which may have negatively affected participants’ baseline mental health. Furthermore, 17.6% of the participants presented moderate depression at T0, a value that decreased to 0% at T4, demonstrating the benefits of the treatment. A study by Bryan J. Kemp^23^ evaluated 120 polio survivors and compared them with 60 healthy individuals, observing a 28% frequency of depression in the PPS group. Additionally, individuals with post-polio syndrome reported a higher average depression score in comparison to non-disabled controls and individuals with polio who either did not experience new symptoms or did experience new symptoms but did not meet the diagnostic criteria for PPS^23^.

Depression has a multifactorial origin. Depression scores have been negatively correlated with family function, health satisfaction, disability acceptance, Instrumental Activities of Daily Living (IADL) score, and PPS status for the sample of polio^23^. Jensen et al.^24^ found a correlation between depressive symptoms and increased pain and fatigue in individuals with post-polio syndrome^24^. Previous research has shown that individuals who experience a decline in their abilities and the onset of additional health problems, especially in clinical settings, may report more severe levels of distress. However, the extent of suffering and depression, as well as the impact of these changes on life satisfaction, are not yet fully understood^23^.

In this study depression, feelings of anxiety and stress were emphasized prior to the REAC therapeutic intervention. At T0, 64.7% of participants reported feeling anxious, with 23.5% reporting extreme anxiety. By T4, this percentage decreased to 6.3%. At the beginning of the study (T0), 76.5% of participants reported feeling stressed, with 29.4% reporting severe stress. By the end of treatment (T4), none of the participants reported severe stress. The study shows that the REAC therapeutic intervention effectively reduces anxiety and stress levels among participants. Additionally, a protocol involving 155 participants who reported mental and physical discomfort due to the Covid-19 pandemic showed a decrease in depression, anxiety, and stress scores after treatment with REAC NPPO-CB Neuromodulation, as measured by the DASS scale^12^. These results are of great importance, particularly given the impact of the COVID-19 pandemic, which has led to a rise in mental health issues in the general population.

PPS is a chronic and progressive condition that results in increasing limitations and challenges over time^25,26^. Dealing with the stress associated with these conditions affects an individual's quality of life and overall well-being. Individuals with PPS face physical limitations related to their clinical condition, including movement, lifting, and transportation^26,27^. Garip et al.^28^ found that post-polio syndrome (PPS) negatively affects quality of life in terms of functional status, pain severity, and energy. Yang et al.^27^ reported that restrictions on mobility and activities have a greater impact on health-related quality of life in polio survivors than in the public, indicating that they have a particularly strong need for ongoing management and support^27^.

The study assessed quality of life using the WHOQoL-bref and found a statistically significant improvement in the physical and psychological dimensions between the T0–T3 and T0–T4 phases. These findings are consistent with Rinaldi et al.^29^ demonstration of the benefits of sequencing treatment protocols for people with Parkinson's disease. The NPO protocol was applied, followed by the NPPO, resulting in an increase in functional capacity and improvements in the mental and physical health components of quality of life.

Research has shown that individuals who have survived polio experience limited mobility and activity, increased pain, and higher levels of depression and anxiety compared to both healthy individuals and the general population with activity limitations^28^. Additionally, sedentary behavior and insufficient physical activity can exacerbate several health problems, both physical and mental, which can directly impact the quality of life of those living with degenerative diseases. This research is significant in the treatment context, particularly considering the scarcity of therapies for this population.

The study demonstrated that REAC treatments had positive effects on pain control, anxiety, stress, depression, and quality of life in the patient group with PPS. Furthermore, it demonstrated an enhancement of the patients' physical and mental health. No side effects were reported with the use of the REAC protocol in this research. Effective pain management is crucial for individuals with PPS, as pain is often related to depression, stress, and anxiety. The REAC neuromodulation protocols offer new insights that can help this patient population manage their pain and improve their overall health. Furthermore, REAC neuromodulation protocols show promise as a complementary intervention for individuals with active lifestyles, offering innovative ways to improve neuropsychological and physical performance in those with PPS. This has the potential to significantly improve overall quality of life.

### Limitation section

REAC treatments may be an effective method for treating post-polio syndrome. However, further studies with larger sample and a study with a control group might provide more conclusive results.

## Conclusion

In conclusion, this study suggests that the REAC NPO, NPPOs, and NMO neuromodulatory treatment protocol hold promise as a valuable therapeutic option for individuals with PPS. The findings suggest that REAC treatments have the potential to improve pain, mental health, and quality of life, and they may represent a safe and effective alternative to traditional treatments for PPS. Future research is needed to further validate the efficacy of REAC neuromodulation treatments for PPS. However, these initial findings suggest that REAC neurobiological treatments have the potential to make a significant contribution to the management of PPS.

## Methods

### Study design

An Open label study was conducted in the Division of Neuromuscular Diseases, Department of Neurology and Neurosurgery, Federal University of São Paulo, São Paulo, Brazil. In accordance with the Declaration of Helsinki^21^ and approved by the Ethics Committee of Federal University of São Paulo—UNIFESP, protocol code 4.526.882.

### Sample size determination and power analysis

To determine the appropriate sample size for our study, we employed sample power analysis using G*Power^30^ (Universität Düsseldorf, Psychologie—HHU). Setting the following parameters: statistical test, Wilcoxon signed-rank test (one-sided), effect size value, 0.5, alpha error probability, 0.05, and power, 0.5, we arrived at a required total sample size of 13 subjects. To account for potential participant dropouts, we opted to augment the study’s patient population.

### Inclusion and exclusion criteria

To minimize the heterogeneity of the study population, we aimed to recruit participants with a relatively consistent clinical presentation.

This inclusion and exclusion criteria were designed to select individuals with a relatively homogeneous clinical profile, minimizing the potential confounding influences of other neurological or systemic conditions.

- *Inclusion Criteria* Confirmed history of poliomyelitis (PV); Established diagnosis of post-polio syndrome (PPS); New onset or worsening of muscle weakness for at least 1 year; Age between 18 and 65 years; Ability to ambulate independently for at least three meters with or without walking aids or orthotics; Preserved verbal communication skills; Adequate cognitive function to understand and consent to study participation.
- *Exclusion Criteria* Severe generalized muscle weakness (Medical Research Council [MRC] grade ≤ 3); Comorbid conditions known to cause neuromuscular dysfunction (e.g., amyotrophic lateral sclerosis [ALS], spinal muscular atrophy [SMA]); Uncontrolled diabetes mellitus; Active alcoholism; Uncontrolled thyroid disease; Multifocal conduction block; Multiple sclerosis; Significant nutritional deficiencies; Active autoimmune diseases; Exogenous intoxication with heavy metals or insecticides.

### Population

The study was realized with 17 PPS patients. The treatments (pharmacological or not) prior to the study were maintained according to the medical advice.

### Procedures and duration of the study

**Figure 4:**
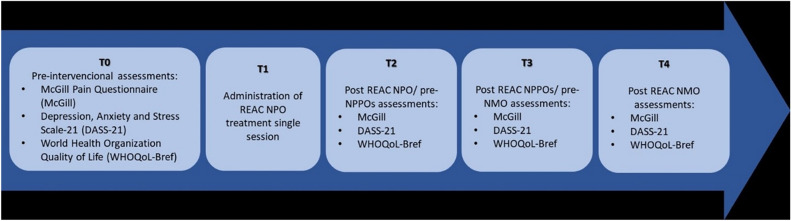
Study flowchart and phases.

Participants were selected based on the inclusion and exclusion criteria. After providing informed consent, the study commenced. Initially, pain levels were assessed using the McGill pain scales, while quality of life was assessed using the WHOQoL-Bref and Depression, Anxiety and Stress Scale (DASS). Subsequently, treatment was administered according to one of four protocols: NPO, NPPO, NPPO-CB, or NMO (Fig. 4).

The protocol spanned a period of 12 weeks, during which assessments and applications were conducted. All evaluations were performed before and after each stage of the research.

### Reproducibility of the study

The device used in this study is the REAC device, model BENE 110, produced by ASMED, Scandicci, Florence, Italy. The administration parameters of each protocol used in this study are preprogrammed into the device and cannot be altered by the operator.

### Interventions—radio electric asymmetric conveyer (REAC) technology

REAC technology is a non-invasive therapeutic approach that harnesses the principles of ultralow-intensity radio electric fields asymmetrically conveyed inside the body to modulate cellular processes^31–33^ and induce therapeutic responses^10,11,29^. It aims to optimize ionic fluxes at the molecular level, enhancing the flow of microcurrents generated by these ion movements. This modulation is believed to influence various cellular activities, including neurotransmission, cell signaling, and cellular metabolism^34–38^.

### Neuro postural optimization (NPO)

NPO is a non-invasive and painless neurobiological modulation technique that utilizes the REAC technology platform to modulate neural activity and promote postural balance and functional integration. The NPO protocol involves a single treatment session lasting several milliseconds, targeting specific neuropathways that influence posture and sensorimotor function^10,11^.

The underlying mechanism of NPO involves modulating the activity of cortical and spinal networks that regulate posture and sensorimotor processing^10,11^. By influencing these neural circuits, NPO aims to enhance postural control, reduce pain, and improve functional outcomes in various conditions associated with postural impairments.

### REAC neuro psycho physical optimization (NPPO) and neuro psycho physical optimization cervico brachial (NPPO-CB)

REAC NPPO^13^ and NPPO-CB^13^ are two non-invasive neurobiological modulation treatments protocols that aim to optimize the body's response to stress and improve overall well-being and physical performance. NPPO and NPPO-CB are based on the principle that the nervous system can be modulated to enhance its ability to regulate and adapt to various stressors.

Patients received NPPO and NPPO-CB simultaneously, with NPPO administered first, followed by NPPO-CB, and the treatment consisted of 18 cycles treatments of each protocol. NPPO-CB was applied immediately following NPPO. This resulted in eight daily applications, four of NPPO and four of NPPO-CB, separated by 1 h.

### REAC neuro muscular optimization (NMO)

REAC NMO is a non-invasive neurobiological modulation treatment to modulate the nervous system and enhance motor control, balance, and physical performance^39^. In order to optimize the management of muscles, in particular the functional balance between agonist and antagonist muscles, both in pathological and dysfunctional conditions.

The treatment plan consists of 10 applications divided into three cycles. The first two cycles consist of four applications per day while the third cycle consists of two applications separated by an hour.

## Instruments assessment

### McGill pain questionnaire (McGill)

The McGill Pain Questionnaire was developed in 1975 by Melzack at McGill University in Montreal, Canada. Its aim was to provide qualitative measures of pain that can be analyzed statistically. It is one of the most widely referenced questionnaires globally and is used in clinical practice. The purpose of this instrument was to evaluate pain across three dimensions: sensory-discriminative, motivational-effective, and cognitive-evaluative, supported by physiologically specialized systems in the central nervous system^40,41^.

The McGill questionnaire was validated for Brazilian Portuguese by Varoli and Pedrazzi in 2006^41^. It assesses the experience of pain in four parts. The first part includes a sketch of a human body to aid in the spatial and depth location of the referred pain. The second section of the questionnaire aims to gather information about the temporal properties of pain, including whether it is constant, periodic, or brief, as well as the circumstances in which painful symptoms begin to be perceived and any analgesic interventions that have been used to alleviate them. The third section is particularly unique as it assists the patient in describing the specific qualities of their pain, with 77 descriptors distributed across 20 subgroups. Patients are instructed to select only one adjective from each subgroup. The given adjectives represent various dimensions of pain, including sensory, affective, evaluative, and miscellaneous categories numbered 1–20. The scores are quantitative, indicating the number of words chosen, and are used to calculate the total pain index, as well as the individual indices for each dimension. The fourth and final section of the questionnaire aims to evaluate the intensity of pain on a numerical scale from 0 to 5. The scale is associated with the following words: 0 for no pain, 1 for mild, 2 for uncomfortable, 3 for distressing, 4 for horrible, and 5 for excruciating^41,42^.

The descriptors used to name pain will depend on the patient’s previous experience and emotional stress caused by their health status, which affects the quality of their pain. Evaluation and scoring will follow the guidelines defined by Melzack R. in 1975, which were obtained through an interview^40^.

#### Depression, anxiety and stress scale-21 (DASS-21)

DASS-21 is a self-report measure of negative emotional state^43–45^. It is a shortened version of the DASS-42, which has been shown to be a reliable and valid measure of depression, anxiety, and stress. The DASS-21 consists of 21 items that are rated on a 4-point scale from 0 (not at all) to 3 (extremely). The scores for each scale are summed to create a total score, with higher scores indicating more severe symptoms^45^.

#### World Health Organization quality of life-bref (WHOQoL-bref)

The WHOQoL—bref assessment, developed by the World Health Organization (WHO)^46^, is a widely acknowledged and extensively employed instrument for evaluating an individual’s perception of their general well-being and quality of life^47^. This assessment provides a comprehensive and multidimensional approach to understanding an individual's physical, psychological, social, and environmental well-being, all of which collectively contribute to their overall quality of life^48,49^.

The questionnaire consists of four domains: physical health, psychological well-being, social relationships, and environment. Each domain contains facets that explore specific aspects. For example, the physical health domain includes facets such as mobility, pain and discomfort, energy and fatigue, and sleep, among others. comprises four domains: physical health, psychological well-being, social relationships, and the environment^50^.

The instrument employs both Likert scale-type questions and open-ended questions to elicit responses from participants. The Likert scale helps quantify subjective experiences, while open-ended questions provide valuable qualitative data, allowing participants to express their thoughts and feelings more freely. This combination enables researchers and healthcare professionals to gain deeper insights into the participants’ perspectives and experiences, thereby enhancing the interpretative validity of the assessment^50^.

Moreover, the WHOQoL assessment is culturally sensitive, acknowledging that quality of life is deeply influenced by cultural norms, beliefs, and values. To ensure its applicability across diverse populations and cultural settings, the WHOQoL questionnaire has been adapted and validated for use in various languages and cultural contexts. This approach fosters cross-cultural comparability and the ability to identify culturally specific aspects that contribute to individuals’ quality of life. In this study we used the Brazilian Portuguese version^51^.

### Statistics analysis

The IBM SPSS^®^ Statistics software, version 23, was employed for conducting the statistical procedures in the present study. Data obtained from the evaluation of REAC treatments were subjected to ANOVA-RM for dependent variables related to DASS-21, McGill, and WHOQoL tests, where participants underwent four conditions: T0, T2, T3, and T4. Bonferroni post-hoc test was used for a posteriori analysis when necessary. The effect size was expressed as ƞ^2^ in all statistical analyses. A significance level of *p* < 0.05 was considered statistically significant.

## Data Availability

Data is provided within the manuscript.
